# Primary colonic well-differentiated / dedifferentiated liposarcoma of the ascending colon: a case report

**DOI:** 10.1186/s40792-017-0373-4

**Published:** 2017-08-30

**Authors:** Hiroshi Sawayama, Naoya Yoshida, Yuji Miyamoto, Tomoyuki Uchihara, Tasuku Toihata, Taisuke Yagi, Yukiharu Hiyoshi, Masaaki Iwatsuki, Yoshifumi Baba, Hideo Baba

**Affiliations:** Department of Gastroenterological Surgery, Graduate School of Medical Sciences, Kumamoto University, 1-1-1 Honjo, Kumamoto, 860-8556 Japan

**Keywords:** Primary colon, Dedifferentiated liposarcoma, Well-differentiated liposarcoma, p16, CDK4, MDM2

## Abstract

**Background:**

Primary colonic and dedifferentiated liposarcomas are both remarkably rare. This work describes a case of primary colonic well-differentiated/dedifferentiated liposarcoma and reviews the clinical characteristics and current therapies for liposarcoma tumors.

**Case presentation:**

A 52-year-old woman was referred to our hospital with a submucosal tumor of the ascending colon. Clinical analysis by ultrasound colonoscopy and computed tomography revealed a partially ossified tumor with irregular edges continuous with the muscular layer. High F-18 deoxyglucose uptake was detected by positron emission tomography. Radical resection with lymph node dissection was performed, yielding a tumor specimen approximately 6.5 × 4.0 × 3.2 cm. Neoplastic spindle cell proliferation was found from submucosa to subserosa. Well-differentiated adipose tissue surrounded the tumor, but contained atypical nuclei with condensed chromosomes. Immunohistochemical staining was positive for p16, CDK4, and MDM2 expression. Based on these findings, a diagnosis of well-differentiated/dedifferentiated liposarcoma was given. Dedifferentiated liposarcomas are more aggressive than their well-differentiated, low-grade counterparts. While local recurrence can occur with both tumor types, dedifferentiated liposarcomas are more prone to developing distant metastases. The patient received no postoperative therapy, and no recurrences have been observed 12 months after surgery.

**Conclusions:**

Here we report an extremely rare case of well-differentiated/dedifferentiated liposarcoma of the ascending colon. The dedifferentiated component was high-grade liposarcoma and well-differentiated liposarcoma was detected around the main tumor.

## Background

Soft tissue sarcomas arise from skeletal or extraskeletal connective tissue in the extremities, retroperitoneum, head and neck, and subcutaneous tissues. Liposarcoma is the single most common soft tissue sarcoma, accounting for at least 20% of all sarcomas and over 50% of retroperitoneal sarcomas [1]. The most recent World Health Organization classification recognizes five categories of liposarcomas: well-differentiated or atypical lipomatous tumor—which includes the adipocytic, sclerosing, and inflammatory subtypes; myxoid; high-grade myxoid; pleomorphic; and dedifferentiated [2]. Primary colonic liposarcoma is rare, while dedifferentiated liposarcoma is even more so. This report describes the surgical resection of a primary colonic well-differentiated/dedifferentiated liposarcoma and discusses the disease characteristics and current treatments.

## Case presentation

**Fig. 1:**
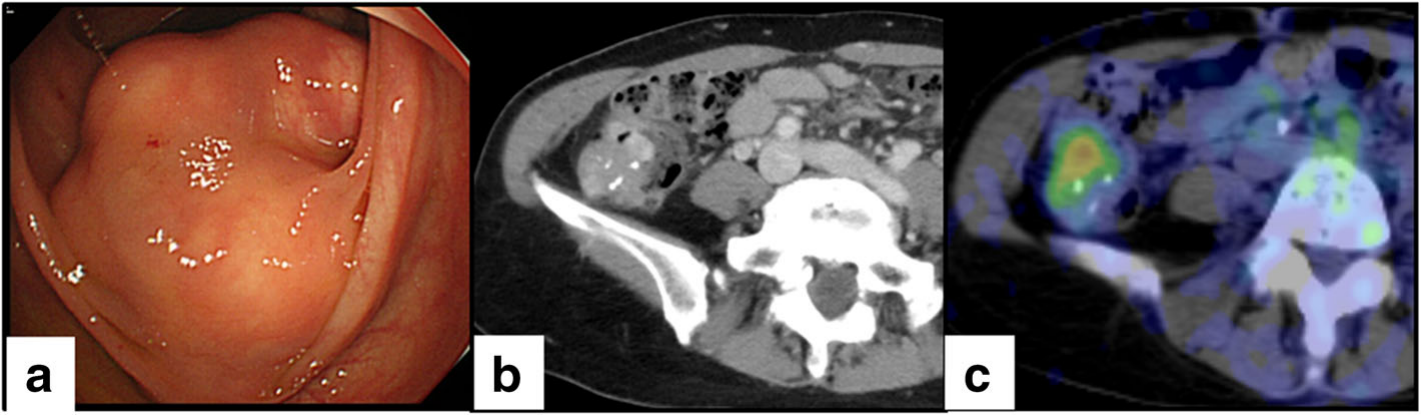
Preoperative examinations. (**a**) A submucosal tumor extending from the liver flexure to the ascending colon was detected by colonoscopy. (**b**) Computed tomography showed irregular tumor edges and highly dense areas indicative of calcification. (**c**) The primary tumor standardized uptake value (SUV_max_) of F-18 deoxyglucose was measured at 2.4–3.4 by positron emission tomography

A 52-year-old woman was referred to our hospital with a submucosal tumor of the ascending colon. The patient voiced no complaints, and no abnormalities were found upon physical examination. She had a past history of pulmonary tuberculosis at 22 years of age. Laboratory findings showed a slight elevation of carcinoembryonic antigen (CEA; 3.6 mg/mL; reference value, <3.4 mg/mL) and other values were normal. Colonoscopy confirmed a submucosal tumor extending from the hepatic flexure to the ascending colon (Fig. 1a), with edges continuous with muscular layer as determined by ultrasound colonoscopy. Biopsy was attempted, but the tissue could not be collected because of bleeding. Computed tomography (CT) revealed irregular tumor edges and highly dense areas indicative of calcification (Fig. 1b). The primary tumor standardized uptake value (SUV_max_) for F-18 deoxyglucose was 2.4–3.4 as determined by positron emission tomography (Fig. 1c). Gastrointestinal stromal or neuroendocrine tumors were considered as a differential diagnosis.

We performed a radical resection with lymph node dissection. General anesthesia was induced with full muscle relaxation, and a skin incision was made in the abdomen. A firm tumor was confirmed in the ascending colon, but the abdominal cavity was devoid of metastatic or disseminated lesions. The ileocolic vein and artery, as well as an accessory right colic vein, were identified, ligated, and cut, and the main lymph nodes on the anterior surface of the inferior mesenteric vein from gastrocolic trunk to ileocolic vein were dissected. We resected along the fusion fascia between the mesocolon and retroperitoneum; the resultant surgical specimen completely encompassed a firm tumor.

**Fig. 2:**
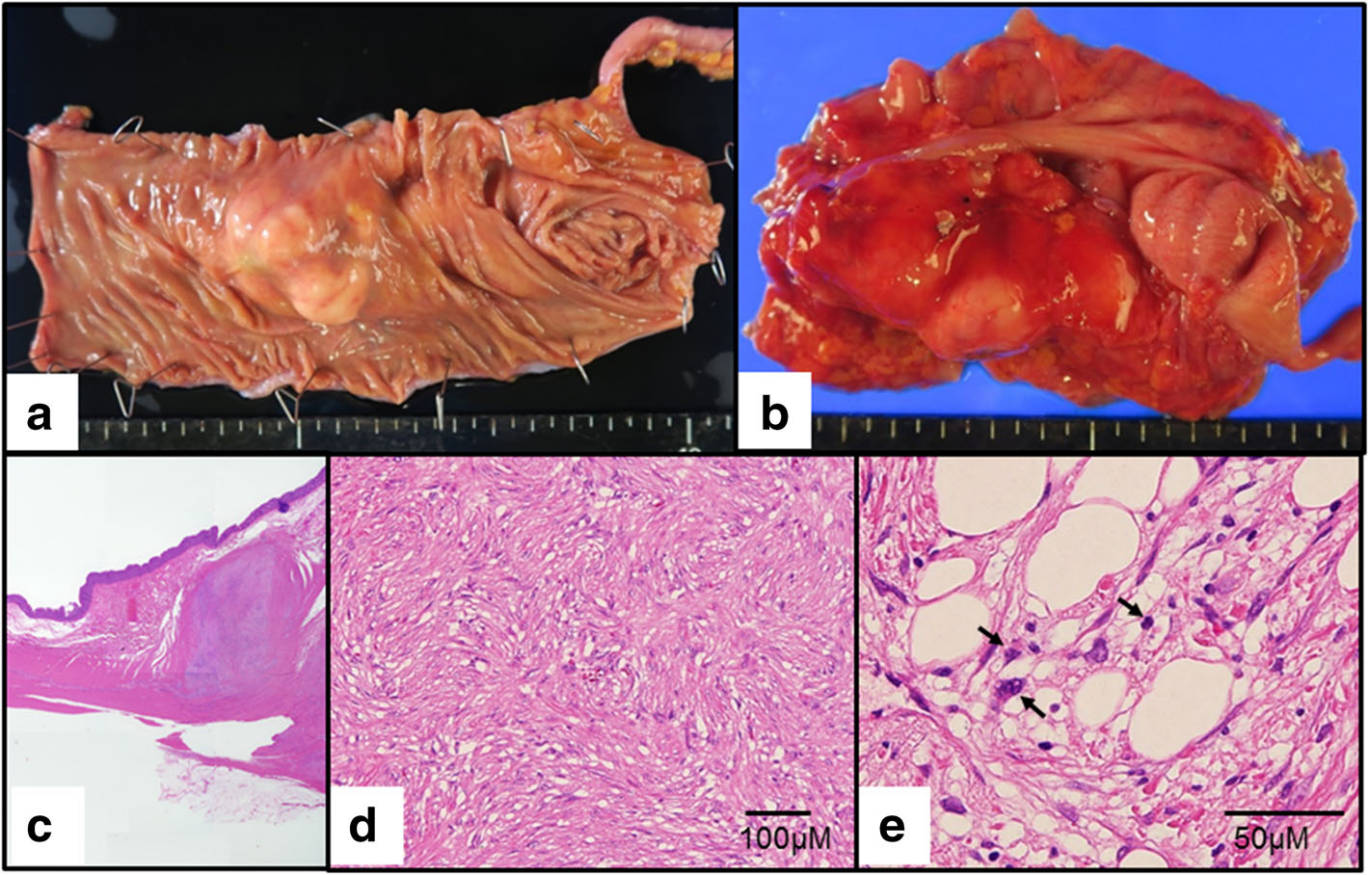
Resected specimen and pathological findings. (**a**, **b**) The resected tumor was 6.5 × 4 × 3.2 cm (83.2 cm^3^). (**c**) Proliferating spindle cells from the submucosa to subserosa. (**d**) Dedifferantiated sarcoma: tumor histopathology revealed proliferating neoplastic spindle cells. (**e**) Well differentiated liposarcoma: atypical nuclei was detected with condensed chromosomes (*arrows*)

**Fig. 3:**
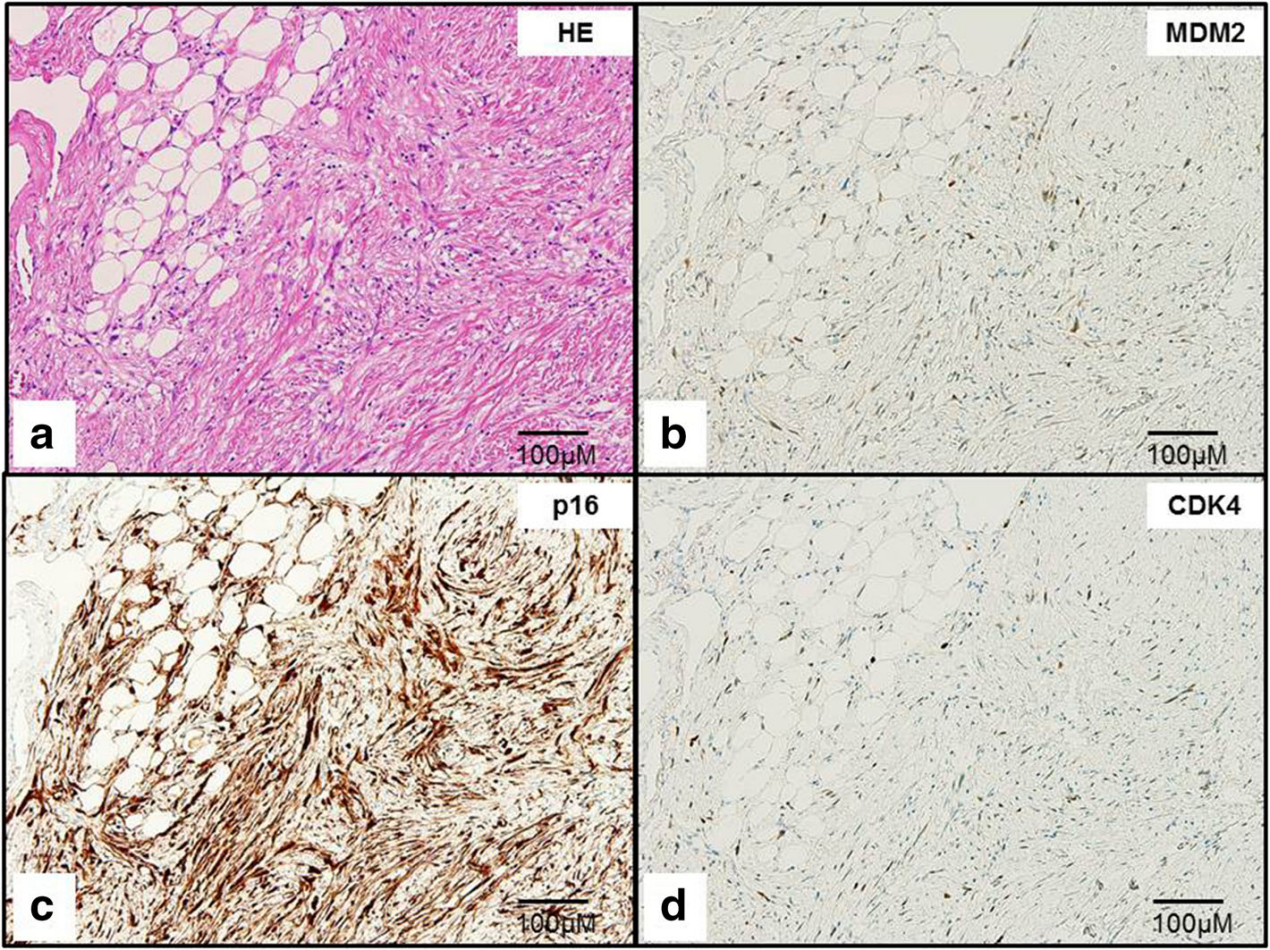
Immunohistochemistry for p16, CDK4, and MDM2 expression. (**a**) Hematoxylin eosin stain. Immunohistochemistry of (**b**) MDM2, (**c**) p16, and (**d**) CDK4

The resected specimen was approximately 6.5 × 4.0 × 3.2 cm (Fig. 2a, b), the center of the solid tumor being located in the middle of the resected specimen. Pathologic examination revealed proliferating spindle cells from the submucosa to subserosa (Fig. 2c, d), as well as partial ossification. The mitotic rate was eight mitoses/10 high-power fields. No necrotic areas were detected, and the tumor was not encapsulated. Well-differentiated adipose tissue surrounded the tumor, but contained atypical nuclei with condensed chromosomes (Fig. 2e). Immunohistochemistry was positive for p16, CDK4, and MDM2 expression (Fig. 3a–d). Based on these clinicopathological findings, the tumor was classified as well-differentiated/dedifferentiated liposarcoma.

A firm mass of dedifferentiated sarcoma was completely resected. However, there were some atypical cells in the adipose tissue around the firm tumor and these were diagnosed as well differentiated liposarcoma. The well-differentiated liposarcoma component extended to the surgical margin in places; however, only normal adipose tissue was detected at most of the surgical margin. A positive surgical margin was detected only for the parts of the well differentiated sarcoma near the firm mass. No lymph node metastasis was detected. Microscopic remnant of well-differentiated liposarcoma was confirmed according to pathological findings, but post-surgical FDG-PET CT and enhanced CT could not detect the remnant tumor around the resected lesion. The patient received no postoperative therapy. We performed follow-up evaluation with enhanced computed tomography or magnetic resonance imaging every 3 months. No recurrences have been observed 12 months after surgery.

## Discussion

This report describes an extremely rare case of ascending colonic well-differentiated/dedifferentiated liposarcoma. PubMed was queried for articles published in English from January 1990 to September 2016 with the terms “liposarcoma” and “colon”. Seventy-six articles were returned, of which 13 and 2 described primary colonic well-differentiated and dedifferentiated liposarcoma, respectively. Primary colonic liposarcoma is rare, and only a few cases of dedifferentiated liposarcoma have been reported [3, 4]; therefore, considerations of the characteristics and treatments of primary colonic liposarcoma generally reference those for retroperitoneal tumors.

Dedifferentiated liposarcomas arise from their well-differentiated counterparts and thus exhibit cytogenetic similarities. Immunohistochemistry for CDK4, MDM2, and p16 expression aids in the differential diagnosis of well-differentiated and dedifferentiated liposarcoma from other adipocytic neoplasms [5]. Despite this, the two display significantly different biologic behaviors. Low-grade, well-differentiated tumors can recur as high-grade, dedifferentiated disease characterized by increased local recurrence rates and metastatic potential. For instance, retroperitoneal well-differentiated and dedifferentiated liposarcomas were associated with 3-year local recurrence rates of 31% and 83%, respectively. Approximately 20–30% of dedifferentiated liposarcomas result in distant recurrence, as opposed to only 3% of well-differentiated tumors—of which all belonged to the sclerosing subtype. Moreover, dedifferentiated liposarcoma is associated with a 4–6-fold increased risk of death when compared with well-differentiated cases [6, 7].

In this case, the dedifferentiated liposarcoma formed a firm mass that was easily identified; however, the well-differentiated sections were soft and difficult to distinguish from the fat tissue surrounding the ascending colon during the operation. We therefore recommend that when dedifferentiated liposarcomas are resected, an adequate margin of soft fatty tissue around the solid tumor also be resected to ensure that no well-differentiated liposarcoma remains. Given that it is difficult to diagnose dedifferentiated liposarcoma preoperatively, submucosal tumors suspected of being high-grade sarcomas need to be resected with adequate margins that include soft tissue around the firm tumor.

Positive margins increase the risk of local recurrence, their influence on overall survival is less clear. Positive microscopic margins (R1) are reportedly associated with higher distant recurrence rates and poorer prognoses than negative microscopic margins (R0) [8], but other studies have failed to demonstrate this relationship [6, 9]. Moreover, 72% of patients with positive margins had no recurrence [8]. Positive margins were poor prognosis than negative margins in the high-grade tumor; however, surgical margins were not related with prognosis in the low-grade tumor [7].

Postoperative treatment for liposarcoma remains controversial. Adjuvant radiotherapy limits the risk of local recurrence, but no definite survival benefit has been established [10] and patients often experience severe adverse effects attributed to cell death in the surrounding normal tissues—primarily the small bowel [11]. In 1054 patients undergoing resection of primary retroperitoneal sarcoma, 276 patients underwent radiotherapy. But the radiotherapy had no significant impact on prognosis (HR, 0.95; 95% CI, 0.78–1.15; *P* = 0.6) [12]. Recent study reported the survival benefits of adjuvant radiotherapy, but the postoperative radiotherapy effect does not depend on surgical margin status (HR 0·93; 95% CI 0·79–1·10; *P* = 0.38) [13]. Comparatively, adjuvant chemotherapy with a doxorubicin-based regimen ((EORTC 62771: doxorubicin, dacarbazine, cyclophosphamide and vincristine; EORTC 62931: doxorubicin and ifosfamide) is an independent favorable prognostic factor for relapse-free, but not overall, survival [14, 15]. In this case, low-grade, well-differentiated liposarcoma were positive but dedifferentiated liposarcoma was completely resected, and no adjuvant therapy was given based on previous studies.

The following recommendations for evaluation during follow-up of retroperitoneal sarcoma have been published by the Trans-Atlantic RPS Working Group. Because the median time to recurrence of high-grade retroperitoneal sarcoma is less than 5 years after definitive treatment, the intervals between follow-up evaluations should be 3 to 6 months for 5 years. After 5 years, annual follow-up is considered adequate. After incomplete resection of retroperitoneal sarcoma patients should be followed indefinitely because the risk of recurrence does not plateau [16].

## Conclusions

This report describes an extremely rare case of ascending colonic liposarcoma consisting of well-differentiated and dedifferentiated sections. Dedifferentiated liposarcomas are high-grade sarcomas; additionally, well differentiated liposarcoma was detected around the main tumor. The dedifferentiated tumor was completely resected, but the aggressive nature of dedifferentiated liposarcoma need periodic examination.

## Abbreviations

CDK4: Cyclin-dependent kinase 4
CEA: Carcinoembryonic antigen
CI: Confidential interval
CT: Computed tomography
FDG: F-18 deoxyglucose
HR: Hazard ratio
MDM2: Murine double minute 2
PET: Positron emission tomography
SUV: Standardized uptake value

## References

[1] Mack TM (1995). Sarcomas and other malignancies of soft tissue, retroperitoneum, peritoneum, pleura, heart, mediastinum, and spleen. Cancer.

[2] Fletcher CBJ, Hogendoorn P, Mertens F. World Health Organization classification of tumours of soft tissue and bone: pathology and genetics of tumours of soft tissue and bone. 4th ed. Lyon, France, IARC Press; 2013.

[3] Turkoglu MA, Elpek GO, Dogru V, Calis H, Ucar A, Arici C (2014). An unusual case of primary colonic dedifferentiated liposarcoma. Int J Surg Case Rep.

[4] Takeda K, Aimoto T, Yoshioka M, Nakamura Y, Yamahatsu K, Ishiwata T (2012). Dedifferentiated liposarcoma arising from the mesocolon ascendens: report of a case. J Nippon Med Sch.

[5] Thway K, Flora R, Shah C, Olmos D, Fisher C (2012). Diagnostic utility of p16, CDK4, and MDM2 as an immunohistochemical panel in distinguishing well-differentiated and dedifferentiated liposarcomas from other adipocytic tumors. Am J Surg Pathol.

[6] Singer S, Antonescu CR, Riedel E, Brennan MF (2003). Histologic subtype and margin of resection predict pattern of recurrence and survival for retroperitoneal liposarcoma. Ann Surg.

[7] Lahat G, Tuvin D, Wei C, Anaya DA, Bekele BN, Lazar AJ (2008). New perspectives for staging and prognosis in soft tissue sarcoma. Ann Surg Oncol.

[8] Stojadinovic A, Leung DH, Hoos A, Jaques DP, Lewis JJ, Brennan MF (2002). Analysis of the prognostic significance of microscopic margins in 2,084 localized primary adult soft tissue sarcomas. Ann Surg.

[9] Zagars GK, Ballo MT, Pisters PW, Pollock RE, Patel SR, Benjamin RS (2003). Surgical margins and reresection in the management of patients with soft tissue sarcoma using conservative surgery and radiation therapy. Cancer.

[10] Mendenhall WM, Zlotecki RA, Hochwald SN, Hemming AW, Grobmyer SR, Cance WG (2005). Retroperitoneal soft tissue sarcoma. Cancer.

[11] Pawlik TM, Ahuja N, Herman JM (2007). The role of radiation in retroperitoneal sarcomas: a surgical perspective. Curr Opin Oncol.

[12] Nathan H, Raut CP, Thornton K, Herman JM, Ahuja N, Schulick RD (2009). Predictors of survival after resection of retroperitoneal sarcoma: a population-based analysis and critical appraisal of the AJCC staging system. Ann Surg.

[13] Nussbaum DP, Rushing CN, Lane WO, Cardona DM, Kirsch DG, Peterson BL (2016). Preoperative or postoperative radiotherapy versus surgery alone for retroperitoneal sarcoma: a case-control, propensity score-matched analysis of a nationwide clinical oncology database. Lancet Oncol.

[14] Le Cesne A, Ouali M, Leahy MG, Santoro A, Hoekstra HJ, Hohenberger P (2014). Doxorubicin-based adjuvant chemotherapy in soft tissue sarcoma: pooled analysis of two STBSG-EORTC phase III clinical trials. Ann Oncol.

[15] Pervaiz N, Colterjohn N, Farrokhyar F, Tozer R, Figueredo A, Ghert M (2008). A systematic meta-analysis of randomized controlled trials of adjuvant chemotherapy for localized resectable soft-tissue sarcoma. Cancer.

[16] Management of primary retroperitoneal sarcoma (RPS) in the adult: a consensus approach from the Trans-Atlantic RPS Working Group. Annals of surgical oncology 2015;22:256–263. 10.1245/s10434-014-3965-2 25316486

